# Spontaneous Intracranial Hypotension: A Commonly Missed Cause of Secondary Headache

**DOI:** 10.7759/cureus.97070

**Published:** 2025-11-17

**Authors:** Farzia Homayra Tanzum, Arun Cherackakudy Joy, Rochan Athreya Krishnamurthy, Shahab Khan, Peter Concannon, Jake Cowen, Dileep Perumala

**Affiliations:** 1 Internal Medicine, Portsmouth Hospitals University NHS Trust, Portsmouth, GBR; 2 Cardiology, Hayatabad Medical Complex Peshawar, Peshawar, PAK; 3 Internal Medicine, Saidu Group of Teaching Hospital, Swat, PAK; 4 Acute Medicine, Portsmouth Hospitals University NHS Trust, Portsmouth, GBR; 5 Radiology, Portsmouth Hospitals University NHS Trust, Portsmouth, GBR

**Keywords:** epidural blood patch (ebp), migraine headaches, orthostatic headache, spontaneous intracranial hypotension (sih), subdural hematoma (sdh)

## Abstract

Spontaneous intracranial hypotension (SIH) is an uncommon but important cause of secondary headache that can closely mimic primary headache disorders, often leading to diagnostic delays. We present the case of a 50-year-old woman with a persistent, treatment-resistant orthostatic headache initially misdiagnosed as migraine, sinusitis, and perimenopausal symptoms. Brain MRI revealed a spontaneous subdural hematoma (SDH) along with classical features of SIH, while spinal MRI demonstrated a small longitudinal epidural fluid collection. Conservative management failed to relieve her symptoms; however, sequential non-targeted epidural blood patches (EBP) resulted in significant clinical and radiological improvement, with complete recovery noted at follow-up. This case underscores the importance of considering SIH in patients presenting with atypical or positional headaches, particularly when accompanied by SDH. Early recognition and appropriate intervention are crucial to prevent complications and optimise patient outcomes.

## Introduction

Headache is one of the most common neurological complaints encountered in clinical practice, with primary headache disorders such as migraine and tension-type headache accounting for the majority of cases. Nevertheless, secondary causes should always be considered, particularly when symptoms are atypical, progressive, or refractory to standard therapies.

Spontaneous intracranial hypotension (SIH) is an uncommon but increasingly recognized cause of secondary headache. SIH refers to a condition in which headaches arise due to reduced cerebrospinal fluid volume or pressure, often linked to spontaneous CSF leakage and confirmed by imaging findings or a low opening pressure on lumbar puncture [1]. The hallmark manifestation is a posture-dependent headache that intensifies within minutes of assuming an upright position, often accompanied by symptoms such as neck discomfort, nausea, vomiting, or photophobia [2]. However, the clinical presentation can be variable and may mimic more common headache disorders, frequently resulting in diagnostic delays [3].

In acute and general medical settings, where headache represents a substantial proportion of neurological presentations, SIH should be considered in patients presenting with new or atypical headaches. We report the case of a 50-year-old woman with a persistent, treatment-resistant headache that was initially misdiagnosed on multiple occasions and was later found to have SIH. This case highlights the diagnostic challenges associated with SIH and underscores the importance of early recognition to prevent potentially serious complications.

## Case presentation

A 50-year-old woman, normally fit and well, presented with a 3-week history of persistent headache. She had a background of infrequent migraine with associated neck pain and perimenopausal symptoms over the past seven months. Her symptoms began the day after performing core-strengthening exercises, initially as upper back pain, followed by a gradual-onset, band-like frontal headache rated 6/10 in severity. The headache worsened on bending forward and partially improved when lying flat. Over-the-counter analgesics provided no relief. There was no history of trauma, head injury, recent travel, smoking, or alcohol use. Family history was notable for migraine in her mother and a brain tumour in her grandfather.

She consulted her general practitioner twice and was first treated with oral antibiotics and a nasal spray for presumed sinusitis, and subsequently with sumatriptan for suspected migraine. Despite treatment, her headache persisted, and her symptoms were later attributed to possible perimenopausal syndrome. One week later, she experienced worsening headache associated with nausea, vomiting, blurred vision, photophobia, and a sense of imbalance, prompting her GP to refer her to the hospital.

On examination, the patient was alert and oriented, with no neck stiffness or focal neurological deficits. Systemic examination was unremarkable, and blood tests were within normal limits. Ophthalmological assessment, including fundoscopy, was normal. She was reassured, safety-net advice was provided, and she was discharged with a provisional diagnosis of atypical migraine or medication-overuse headache. Due to the atypical features and absence of a definitive diagnosis, an outpatient CT brain was requested to exclude intracranial pathology.

**Figure 1:**
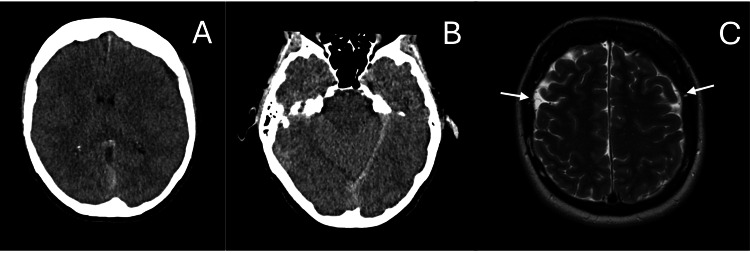
Axial non-contrast CT brain demonstrating a shallow left parafalcine subdural hematoma (A) with extension to the left tentorium cerebelli (B). T2-weighted axial MRI sequence showing bilateral shallow subdural collections (C) (white arrows).

CT brain imaging revealed a 6 mm left frontal subdural collection without mass effect. Given the unexpected finding of an SDH in a relatively young woman, she was recalled to the hospital the following day for further evaluation. A repeat CT brain with CT angiography confirmed a small left parafalcine SDH extending to the tentorium (Figures 1A-1B), along with a mild left frontal subdural haematoma. No vascular malformations or aneurysms were identified. Neurosurgical consultation was obtained; surgical intervention was not recommended due to the uncomplicated nature of the bleed. However, a neurology referral was advised to investigate alternative causes of headache, including reversible cerebral vasoconstriction syndrome (RCVS).

Neurology assessment revealed no focal neurological deficits; however, in light of the unexplained subdural bleed and history of orthostatic headache following exercise, SIH was considered the leading differential, and RCVS was deemed unlikely. Other possibilities, including cerebral venous sinus thrombosis (CVST) and transformed migraine, were also considered. Conservative management was initiated, including strict bed rest, hydration, oral caffeine, and analgesia, while awaiting further imaging.

**Figure 2:**
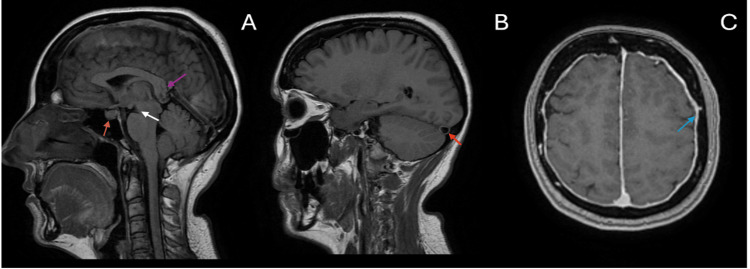
Sagittal T1 FLAIR images (A, B) demonstrating signs of intracranial hypotension, including drooping of the corpus callosum (pink arrow), midbrain sagging with reduced mamillopontine distance (3.5 mm) (white arrow), a bulky pituitary gland (orange arrow), and an engorged venous sinus (red arrow). Axial T1 post-contrast sequence (C) showing diffuse dural thickening and enhancement (blue arrow). FLAIR: Fluid-attenuated inversion recovery.

**Figure 3:**
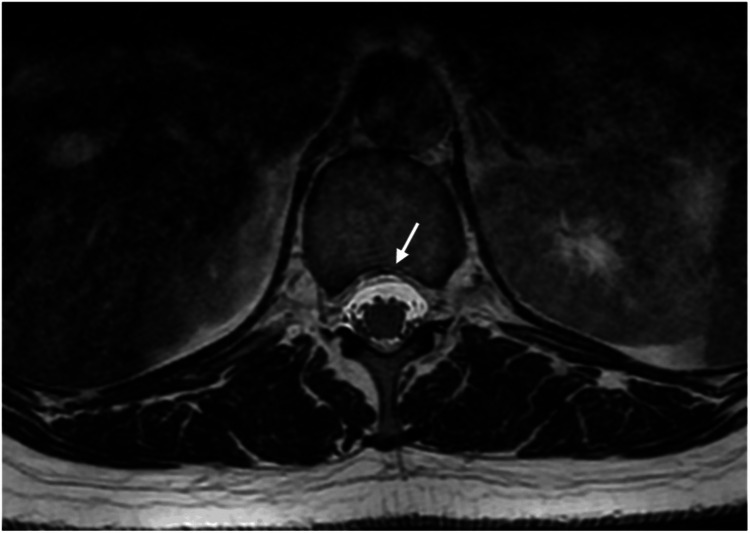
Axial T2 Cube sequence demonstrating a shallow anterior epidural CSF collection (white arrow) extending from T12 to L1.

MRI and MRV of the brain demonstrated a shallow subdural collection (Figure 1C) without mass effect. Additional findings included bilateral dural thickening, an enlarged pituitary gland, brainstem sagging, drooping of the corpus callosum, reduced mamillopontine distance, and venous sinus engorgement, all consistent with SIH (Figure 2). No evidence of CVST was identified. MRI of the spine revealed a small anterior longitudinal epidural collection (SLEC) between T12 and L1 (Figure 3).

Based on these findings, a diagnosis of SIH secondary to SLEC was made. As conservative management failed to relieve her symptoms, the patient underwent a non-targeted lumbar epidural blood patches (EBP) with 12 mL of autologous blood, resulting in partial improvement in headache and visual symptoms. However, symptoms recurred within days, prompting a second non-targeted lumbar EBP with 15 mL of blood four weeks later.

**Figure 4:**
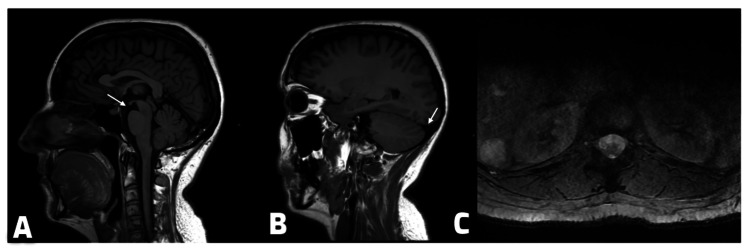
Follow-up imaging approximately 10 weeks after the first MRI. Sagittal T1 FLAIR brain images showing reduced brainstem sagging with an increased mamillopontine distance (6.5 mm) (A) and reduction in venous sinus engorgement (B). Axial 2D MERGE spine sequence demonstrating resolution of the anterior epidural collection (C). FLAIR: Fluid-attenuated inversion recovery; MERGE: Multi-Echo Recombined Gradient Echo.

Follow-up MRI of the brain and whole spine, performed approximately 10 weeks after the initial MRI, demonstrated significant resolution of several SIH features, with no evidence of brain sagging or venous engorgement and a normal spinal study (Figure 4).

At clinical follow-up 11 weeks after the second procedure, the patient reported marked symptomatic improvement and had returned to work and resumed regular exercise, including swimming.

## Discussion

SIH remains an underrecognized clinical entity with an incompletely understood pathophysiology, and delayed diagnosis may result in serious or potentially life-threatening complications [3]. The condition is believed to arise from a loss of spinal CSF volume, which reduces the brain’s supportive buoyancy in the upright posture. This results in downward sagging of intracranial structures, stretching of the dural membranes, particularly in the posterior fossa, and secondary venous distension due to compensatory vascular changes [4]. The annual incidence appears to be higher than previously estimated [5].

Risk factors 

A variety of factors have been implicated in the development of SIH, including spinal trauma related to surgical procedures and lumbar puncture. Activities involving sudden or excessive spinal movement, such as yoga and Pilates, have also been identified as possible precipitating events. However, most patients with SIH cannot identify a definite trigger. In a study of 80 consecutive patients with SIH, only 28 reported an event coinciding with symptom onset, and most of these were atraumatic [3]. In our patient’s case, symptoms began after a session of weighted core exercises. Although weighted core workouts are not commonly described as triggers, similar activities such as yoga, Pilates, and even weightlifting have been associated with spontaneous dural tears and CSF leaks. It is therefore reasonable to consider her workout as a potential precipitating factor [6-8].

Presentation**

In this case, the patient clearly exhibited the hallmark symptom of SIH, an orthostatic headache, yet her symptoms were initially attributed to sinusitis, migraine, and perimenopausal changes in primary care, resulting in delayed diagnosis and worsening symptoms. Such diagnostic delay is not uncommon in SIH. Schievink WI described how initial misdiagnoses frequently included migraine, meningitis, posterior cervical strain, subarachnoid haemorrhage, psychogenic headache, and even malingering, contributing to significant delays in diagnosis and potential complications [3]. Migraine was identified as the most common misdiagnosis in that study. Since the patient's presentation was inconsistent with typical migraine characteristics, which often involve a gradual onset, unilateral, pulsating pain associated with nausea, photophobia, and phonophobia, and are not typically positional, migraine was excluded as a differential diagnosis in this case.

This case underscores the need for vigilance regarding red flags that may indicate a secondary headache, particularly in patients with acute changes in a previously diagnosed primary headache disorder. Recognising when the clinical picture no longer aligns with a benign pattern is crucial to preventing diagnostic delays. The SNNOOP10 mnemonic, which lists red and orange flags associated with headaches, is a valuable clinical tool for identifying warning features that warrant further investigation. It provides a structured approach to headache assessment and assists in guiding decisions regarding advanced imaging, such as MRI or CT, to rule out serious underlying pathology [9]. As a qualitative clinical tool, the presence of any red flag in the SNNOOP10 list should prompt consideration of a secondary headache.

Diagnosis 

The diagnosis of SIH primarily relies on clinical suspicion supported by cranial magnetic resonance imaging and myelography. Several diagnostic criteria have been established to facilitate accurate identification of SIH. Among these, the International Classification of Headache Disorders, 3rd edition (ICHD-3) criteria provide a standardised framework for diagnosis (Table 1). Our patient met all the outlined criteria, confirming the diagnosis of SIH [10].

**Table 1:** Diagnostic criteria for spontaneous intracranial hypotension (based on ICHD-3). SIH: Spontaneous intracranial hypotension; ICHD-3: International Classification of Headache Disorders, 3rd edition.

Criterion	Description
A	Any headache fulfilling criterion C. Typically orthostatic (worsens on standing, improves lying down), but this is not mandatory.
B	Either or both of the following: Low CSF pressure (<60 mm H₂O); Imaging evidence of CSF leakage (e.g., brain sagging, pachymeningeal enhancement, or extradural CSF on spinal imaging)
C	No history of a procedure or trauma known to cause CSF leakage.
D	Headache develops in temporal relation to low CSF pressure or CSF leak, or leads to its discovery.
E	Not better explained by another ICHD-3 diagnosis.

In the absence of trauma, CT imaging revealed a unilateral subdural haematoma. Such haematomas are recognised, though sometimes deceptive, manifestations of SIH. In a 2014 Journal of Neurosurgery cohort, 20 of 27 nongeriatric patients with chronic subdural haematomas linked to spinal CSF leaks had unilateral collections, whereas only seven had bilateral involvement. Our patient’s unilateral haematoma aligns with this pattern, although bilateral cases have also been described [11]. It is important to note that the detection of SDH frequently prompts neurosurgical evaluation, which may paradoxically exacerbate the patient’s condition [12]. In this case, while the patient was referred to neurosurgery for SDH management, no surgical intervention was recommended. Instead, referral to neurology was advised to explore alternative diagnoses given the spontaneous aetiology of the haemorrhage.

Following neurological consultation, SIH was prioritised as the leading differential diagnosis, based on the patient’s characteristic orthostatic headache, the presence of red-flag symptoms, and a spontaneous unilateral subdural haematoma. Although RCVS was initially considered by the neurosurgical team, it was later excluded due to the absence of recurrent thunderclap headaches, a normal CT angiogram, and MRI findings that demonstrated classical features of SIH consistent with the clinical presentation. This clinical reasoning supported the initiation of conservative management even before definitive imaging confirmation. MRI of the brain demonstrated hallmark features of SIH, including subdural fluid collections, pachymeningeal enhancement, engorged venous structures, pituitary hyperaemia, and brain sagging [13]. Further evaluation with spinal MRI revealed a small anterior SLEC, thereby supporting the diagnosis. Although not performed in our patient, myelography remains the imaging modality of choice for localising spinal CSF leaks in SIH [3].

Treatment 

Treatment options once a CSF leak is confirmed include conservative management, non-targeted EBP, targeted EBP, and surgery. Conservative therapy should be discussed with all patients and can be implemented for up to two weeks from symptom onset; this includes bed rest, hydration (2.0-2.5 L daily), and oral caffeine. However, success rates are modest, as shown by several studies [13].

If conservative management fails, EBPs constitute the next line of treatment for SIH. The proposed mechanism by which EBPs facilitate leak closure involves the interaction between injected blood and procoagulant components within the CSF at the dural defect, providing a scaffold for healing. Additionally, cephalad displacement of CSF may reduce pressure at the leak site, thereby promoting closure [14]. A recent multidisciplinary consensus guideline, developed by a 29-member specialist group focused on SIH diagnosis and management, recommends administering at least two non-targeted EBPs spaced two to four weeks apart if the initial attempt is unsuccessful, following a maximum of two weeks of conservative therapy. If symptoms persist, advanced imaging with CT or MR myelography is advised to localise the leak and guide targeted EBP or surgical intervention [15]. Despite initial improvement, symptom recurrence can occur. In one cohort of 23 patients, 41.7% experienced symptom recurrence after initial relief with EBP, with 80% of these responding successfully to a second EBP [16]. This highlights the complexity of SIH and the need for ongoing monitoring. In our case, the patient’s symptoms did not improve following conservative therapy and the first non-targeted EBP but showed significant resolution after the second non-targeted EBP at four weeks, obviating the need for myelography. Early treatment of SIH is associated with a favourable long-term prognosis [17], as demonstrated by our patient who achieved complete neurological recovery.

Complications 

Delayed recognition of SIH can result in significant morbidity, most notably the development of subdural fluid collections and chronic daily headache syndromes. Schievink highlighted that such complications often arise when SIH is misdiagnosed as migraine or tension-type headache, leading to inappropriate treatment and prolonged disability. This emphasises the importance of early identification and intervention, as demonstrated in our case, where timely consideration of SIH helped prevent progression to more severe sequelae [3].

## Conclusions

This case highlights the diagnostic challenges posed by SIH, a frequently under-recognized cause of secondary headache that can mimic more common primary headache disorders. The presence of atypical features, such as an orthostatic headache and an SDH without a history of trauma, should prompt clinicians to consider SIH early in the diagnostic process. Timely recognition and appropriate imaging are crucial to avoid misdiagnosis and prevent potentially serious complications. While conservative management remains the initial approach, EBP plays a pivotal role in symptom resolution, particularly when conservative measures fail. Our patient’s favourable outcome following a second EBP underscores the importance of persistence in treatment before progressing to more invasive diagnostics or surgery. Increasing awareness of SIH among clinicians will improve diagnostic accuracy and patient outcomes in this potentially reversible condition.
